# Differences in the prescribing of potentially inappropriate medicines in older Australians: comparison of community dwelling and residential aged care residents

**DOI:** 10.1038/s41598-020-66991-x

**Published:** 2020-06-23

**Authors:** Hosam Bony, Renae A. Lloyd, Elizabeth D. Hotham, Lauren J. Corre, Megan E. Corlis, Helen A. Loffler, Gregory K. Scarlett, Jacquie M. Tsimbinos, Ian P. Todd, Vijayaprakash Suppiah

**Affiliations:** 1 UniSA Clinical and Health Sciences, University of South Australia, Adelaide, SA Australia; 2 Helping Hand Organisation, Adelaide, SA Australia; 3 Haddad Pharmacy Group, Adelaide, SA Australia; 4 TerryWhite Chemmart Pharmacy, Adelaide, SA Australia; 5 Pharmacy Guild of Australia, South Australian Branch, Adelaide, SA Australia; 6 Australian Centre for Precision Health, University of South Australia, Adelaide, SA Australia; 7 Discipline of Pharmacology, Adelaide Medical School, University of Adelaide, Adelaide, SA Australia

**Keywords:** Risk factors, Geriatrics

## Abstract

Potentially inappropriate medications (PIMs) can contribute to morbidity through exacerbations or progression of existing conditions among older people. In order to characterize the prevalence of PIMs according to the Beers Criteria in older Australians, three hundred and eleven participants were recruited from three residential aged care facilities (RACFs) and two hundred and twenty participants from three community pharmacies in South Australia for a retrospective audit of medication administration charts and community pharmacy dispensing histories. Although a similar number of participants were prescribed at least one PIM (P = 0.09), the average number of PIMs was significantly greater in the RACF cohort (1.96 vs 1.26, P < 0.05). Additionally, PIMs prescribed as *pro re nata* (PRN) in the RACF cohort had a significantly low administration rate compared to prescription rate (19.7% vs 40.7%). The mean number of PIMs within each cohort was statistically significant (RACF = 1.93 vs CDOA = 1.26, P < 0.05). RACF residents were at a slightly greater risk of being prescribed more than one PIM compared to those within the community. Routine medication reviews by pharmacists embedded in RACFs and within the community could be utilised to detect PIMs before such harm occurs.

## Introduction

Medication prescribing and management in older people can be complex, with longer life expectancies, and the high rate of chronic disease amongst Australians over 65 years all contributing to increasing polypharmacy^1,2^. Additionally, altered pharmacokinetics and pharmacodynamics of medications make the elderly more susceptible to adverse effects of medications and heighten sensitivity to therapeutic effects^3–5^.

The use of five or more medications is generally accepted as the definition of polypharmacy^6^. However, the concept is not restricted to number of medications, but also includes the use of unnecessary and inappropriate medications^7,8^. A significant association has been described between the number of medications prescribed and the likelihood of potentially inappropriate medications (PIMs)^9^.

Under-prescribing, mis-prescribing or over-prescribing^10,11^ can all be described as inappropriate, with instances including: medications that cause more harm than benefit, prescribing at a frequency, duration or dose above or below what is recommended, medications with a high risk of drug interactions, therapeutic duplications and omissions of clinically indicated medications^12,13^.

Although on occasions polypharmacy is appropriate, it may still increase the potential for drug interactions and adverse drug reactions, which can contribute to low medication adherence, high healthcare costs and poorer quality of life for older people^14–17^. Many older Australians are on multiple medications and their continued use may not be rationalised, despite successful precedents^18,19^.

Achieving the desired balance between benefits and risks of medication use in multi-morbid clients is important^20^ but challenging, as evidence-based clinical guidelines generally focus on treatment of a single disease or disorder^21^.

Various tools have been developed to aid screening for PIMs^13,21–24^. The first explicit tool to be developed was the Beers Criteria in 1991^22^, which has since been updated several times.

## Aim of Study

The study objective was to characterise the presence of PIMs according to the AGS 2015 Beers Criteria^13^, across one cohort of residential aged care facilities (RACFs) and one cohort of community dwelling older Australians (CDOA), aged 65 years and above.

## Method

In this cross-sectional study, medication histories from medical administration charts were transcribed for RACF residents (June to August 2015 and February to April 2016) who had been living in RACFs for more than 4 weeks. Dispensing data of CDOA were transcribed from dispensing databases belonging to three metropolitan community pharmacies (June to August 2017). The RACFs and the community pharmacies owned the data, which were de-identified at collection; no participant was interviewed.

For RACF residents, apart from demographics and comorbidities (if available), data were collected on the following medication-related factors: medication name, dose, frequency of administration, route of administration, and duration of treatment within the past month (categorised for regular medicines as less than 2 weeks or between 2 and 4 weeks). For “when required” (PRN) medications, the number of administrations over one month was recorded. Administration was documented as regular, PRN, “immediate” (STAT) or short term; indication (if available), prescriber(s) and the pharmacy supplying medications were also recorded. The same medication data were collected in the community; however, adherence and quantification of PRN medication use could not be confirmed. The Anatomical Therapeutic Chemical (ATC) Classification System was used to categorise all recorded medications and assist in analysis^25^.

The AGS Beers Criteria 2015 tool was used to assess PIMs, independent of diagnosis or medication dose^13^. This tool provides an explicit list of PIMs best avoided in older adults and in those with certain diseases or syndromes, as these medications have been found to be associated with poor health outcomes, including falls and even mortality^13,26–28^.

For the RACF participants, *prescription* of a PIM, as identified by the Beers Criteria, was recorded if it was written on the medication chart; however, *exposure* to a PIM was only registered if a participant was actually administered that medicine. Hence, although PRN medications prescribed were included in the total count of prescribed PIMS, further analysis was used to quantify the PRN PIMS actually administered. For the community-dwelling participants, if the participant was dispensed a PIM, he or she was considered exposed, even though self-administration, of either a regular or a PRN medication, could not be confirmed.

Table 2 of Beers Criteria 2015^13^ was used to identify specific PIMs; however, individual participant comorbidities and treatment durations were not taken into account. Table 3 of the Beers Criteria 2015^13^ was used to identify PIMs for the RACF participants based on participants’ comorbidities, but, as comorbidities of the community clients were not available, that group had to be omitted from this analysis.

**Table 1:** Demographics and prescription patterns of the two cohorts in the study population.

Parameter	Residential aged care (RACF) population (n = 311)	Community dwelling older Australians (CDOA) (n = 220)	P value (total RACF vs CDOA) (OR, 95% CI)
Gender (%)			
Female	228 (73.3)	129 (58.6)	**<0.001**^
Male	83 (26.7)	91 (41.4)	
Mean age, years (range)	85.9 (66–104)	79.6 (65–97)	<0.001*
Total number of **regular medications** prescribed (mean) [range]	2870 (9.2)[0–25]	1575 (7.2)[0–16]	0.73*
Number of participants prescribed the following absolute number of medications [%]			**<0.001**^**a^**^ **(9.29, 3.83–22.56)**
0	0	0	
1–4	6 (1.9)	34 (15.5)	
5–9	68 (21.9)	114 (51.8)	
10–14	125 (40.2)	57 (25.9)	
15–19	80 (25.7)	14 (6.4)	
20–24	21 (6.8)	0	
>25	11 (3.5)	1 (0.5)	
**Number of participants prescribed the following number of PIMs [%]**			
0	52 (16.72)	50 (22.72)	0.09^**^**^
1	77 (24.76)	95 (43.18)	**<0.001**^**^**^ **(3.00, 2.00–4.48)**
2	84 (27.01)	51 (23.18)	0.67^**^**^
3	56 (18.00)	17 (7.73)	**<0.01**^**^**^ **(0.40, 0.23–0.72)**
4	26 (8.36)	6 (2.73)	**<0.05**^**^**^ **(0.33, 0.13–0.81)**
5	13 (4.18)	1 (0.45)	**<0.05**^**^**^ **(0.11, 0.01–0.86)**
6	3 (0.96)	0	—
Total participants with PIMs	259 (83.28)	170 (77.27)	0.09^**^**^
Total number of PIMS	600	277	**<0.05**^**^**^
Mean number	(1.93)	(1.26)	
Range	[0–6]	[0–5]	

^*a*^Comparison of polypharmacy (participants on 4 or less medications vs on 5 or more medications). ^P values are obtained from two-tailed Fisher Exact, *P values from Mann Whitney U test.

**Table 2:** AGS 2015 Beers Criteria Table 2 Comparison – Potentially Inappropriate Medication Use in Older Adults.

Therapeutic Category	Residential Aged Care Facility cohort with PIMs (n = 259)^a^	Community cohort with PIMs (n = 170)	P value (PIM in RACF vs PIM in CDOA) (OR, 95% CI)^b,c^
	Frequency of PIM prescribed (% of 259)	Frequency of PIM prescribed (% of 170)	
**Anticholinergics**	5 (1.93)	1 (0.59)	0.41
**Antiparkinsonian**	1 (0.39)	4 (2.35)	0.08
**Antispasmodic**	9 (3.47)	0	—
**Antithrombotics**	1 (0.39)	0	—
**Anti-Infective**	12 (4.63)	2 (1.18)	0.05
**Cardiovascular**	41 (15.83)	18 (10.59)	0.15
**Central Nervous System**			
Antidepressants	17 (6.56)	21 (12.35)	**0.05 (2.0, 1.03–3.93)**
Antipsychotics	93 (35.91)	20 (11.76)	<**0.001 (4.20, 2.47–7.15)**
Benzodiazepines	173 (66.80)	66 (38.82)	<**0.001 (3.17, 2.12–4.74)**
Non-Benzodiazepines	0	3 (1.76)	—
**Endocrine**	2 (0.77)	9 (5.29)	**<0.01 (7.18, 1.53–33.67)**
**Gastrointestinal**			
(Other than Proton Pump Inhibitors)	99 (38.22)	4 (2.35)	<**0.001 (25.68, 9.23–71.42)**
Proton Pump Inhibitors	137 (58.90)	116 (68.24)	<**0.01 (1.91, 1.28–2.87)**
**Pain Medications**			
Non-Selective NSAIDs	10 (3.86)	8 (4.71)	0.81
**Genitourinary**	0	1 (0.59)	—
**Total**	600	277	

^a^Residents with both regular and PRN orders for the same medication were recorded as only 1 PIM. ^b^P values are obtained from two-tailed Fisher Exact probability test. ^c^OR and 95% CI expressed according to increased risk.

**Table 3:** Prescription and administration data for PIMs (both regular and PRN) in the residential aged care population.

Therapeutic Category	Residential Aged Care Facility cohort with PIMs (n = 259)a			
	Frequency of total PIM prescribed (% of 259)	Frequency of regularly administered PIM (% of total prescribed)	Frequency of total PRN PIM prescribed (% of total prescribed)	Frequency of PRN administered PIM (% of PRN PIM prescribed)
**Central Nervous System**				
Antidepressants	17 (6.56)	17 (100.0)	0	0
Antipsychotics	93 (35.91)	67 (72.04)	26 (27.96)	2 (7.69)
Benzodiazepines	173 (66.80)	77 (44.51)	96 (55.49)	24 (25.0)
Endocrine	2 (0.77)	1 (50.0)	1 (50.0)	0
**Gastrointestinal**				
(Other than Proton Pump Inhibitors)	99 (38.22)	6 (6.06)	93 (93.94)	10 (10.75)
Proton Pump Inhibitors	137 (58.90)	133 (97.08)	4 (2.92)	1 (25.0)
Total	600	356 (59.3)	244 (40.7)	48 (19.7)

^a^Residents with both regular and PRN orders for the same medication were recorded as only 1 PIM.

The University of South Australia’s Human Ethics Committee (31911 and 35814), the institutional ethics committee of the RACF organisation and the owners of the three community pharmacies granted approval for this study. All methods were performed in accordance with that Ethics Committees’ relevant guidelines and regulations. As the RACF organisation and the owners of the three community pharmacies owned the data that were used in this study and they gave consent to use the data, RACF residents and community dwelling participants were not approached for individual consent. Additionally, as this study was a secondary analysis of routine collected de-identified data, written and informed consent from individual participants were not deemed necessary in the Ethics Committees’ approvals.

Statistical analysis was conducted using SPSS v.21® and Microsoft Excel®. Firstly, comparisons of number of regular medications and PIMs were made between the two cohorts. Next, differences in therapeutic classes between the two cohorts were compared. Since, administration data was collected in the RACF cohort, the difference between prescribed and administered PRN PIMs was made. Lastly, the Beers Criteria was used to identify PIMs based on each RACF resident’s comorbidities. All categorical data were analysed via the two-tailed Fisher exact test or chi squared test while the Mann-Whitney U test was used to analyse non-categorical data.

## Results

Three hundred and eleven RACFs residents and two hundred and twenty community dwellers were included in this study. Even though, ninety-five RACF participants had a documented diagnosis of dementia, there was no statistical difference (P = 0.124) between the number of regular medications for RACF residents with or without dementia. Hence, all analyses were conducted with the RACF cohort as a whole.

Of the 531 study participants, 67.2% were females. The RACF group was significantly older (85.9 years vs 79.6 years, P < 0.001), and had more females (P < 0.001) than the CDOA cohort (Table 1).

On average, RACF participants were prescribed more regular medications than community dwellers (9.2 vs 7.2 respectively, P = 0.73). However, polypharmacy (≥ five medications) was nine times more in the RACF group (P < 0.001, OR = 9.29 95% CI = 3.83–22.56) (Table 1).

Table 1 also highlights both the number of participants prescribed a specific quantity of PIMs and the total number of PIMs prescribed in each cohort. Although the number of individuals in the RACF cohort with at least one PIM was marginally higher than for the CDOA (83.3% vs 77.3% respectively), this difference was not statistically significant (P = 0.09). However, the mean number of PIMs within each cohort was statistically significant (RACF = 1.93 vs CDOA = 1.26, P < 0.05). After excluding individuals without any PIMs from both cohorts, significant differences were also observed when comparing those receiving one, three, four and five PIMs (P < 0.001, OR = 3.00, 95% CI = 2.00–4.48; P < 0.01, OR = 0.40, 95% CI = 0.23–0.72; P < 0.05, OR = 0.33, 95% CI = 0.13–0.81 and P < 0.05, OR = 0.11, 95% CI = 0.01–0.86 respectively) (Table 1). Community dwellers were more likely to be prescribed only one PIM, as compared to residents from the RACF cohort who were more likely to be prescribed three to five PIMs.

In total, there were 600 PIMs for the RACF residents and 277 PIMs for the CDOA group. The most common medications administered to RACF residents were for symptomatic relief of conditions such as constipation, pain, insomnia, and mood or behavioural issues (Table 2). By contrast, the community cohort was more likely to be prescribed medications for the prevention of morbidity and/or mortality associated with chronic diseases, such as lipid-modifying, anti-hypertensive and acid-related gastrointestinal disorders (Table 2).

Table 2 shows that RACF participants were more likely to be prescribed: antipsychotics (35.91% vs 11.76%, P < 0.001, OR = 4.20 95% CI = 2.47–7.15), benzodiazepines (66.80% vs 38.82%, P < 0.001, OR = 3.17 95% CI = 2.12–4.74) and gastrointestinal agents other than proton pump inhibitors (PPIs) (38.22% vs 2.35%, P < 0.001, OR = 25.68 95% CI = 9.23–71.42). CDOA were more likely to be prescribed: antidepressants (12.35% vs 6.56%, P = 0.05, OR = 2.00 95% CI = 1.03–3.93), endocrine agents (mainly oestrogens) (5.29% vs 0.77%, P < 0.01, OR = 7.18 95% CI = 1.53–33.67) and PPIs (68.24% vs 58.90%, P < 0.01, OR = 1.91 95% CI = 1.28–2.87).

For the RACF participants, administration data (regular and PRN) were also collected over the 4 week study period. Table 3 shows the administration data for those PIMS with significantly different prescribing rates between the two cohorts as identified in Table 2. Overall 59.3% (356 of 600) of PIMs were prescribed and administered as a regular order on the medication chart. Of the remaining 244 (40.7%) prescribed as PRN medications, only 48 (19.7%) were administered. Therefore, of the total 600 PIMs prescribed for this cohort, 32.6% were not administered.

Lastly, the Beers Criteria 2015 was used to identify PIMs on the basis of each RACF resident’s comorbidities. Table 4 summarises the documented comorbidities with the greatest percentage of residents prescribed a PIM. The highest frequency of PIMs use according to disease/syndrome for the RACF participants correlated with a documented history of falls and fractures (n = 61) with 62.3% administered a PIM, followed by those with a diagnosis of dementia or cognitive impairment (n = 95) with 46.3% administered a PIM. Residents with documented falls and fractures were most commonly prescribed opioids and benzodiazepines, which may increase their risk of falls; residents with dementia were administered benzodiazepines and antipsychotics, which may contribute to worsening cognition. Further, antipsychotics also carry a high risk of mortality and stroke in this population^13^. Lastly, out of ten residents with Parkinson’s disease, two were prescribed a PRN dopamine antagonist antiemetic (metoclopramide or prochlorperazine), drugs with potential to worsen Parkinsonism. The overall prescribing of these PRN medications was low and they had not actually been administered; however, they had been prescribed for 20% of residents with Parkinson’s disease, which is not an acceptable pharmacotherapeutic combination.

**Table 4:** AGS 2015 Beers Criteria for PIM use in older adults due to drug-disease or drug-syndrome interactions that may exacerbate the disease or syndrome in the RACF cohort.

Current Disease State (n = Total Count with Disease)	Medication	No. Prescribed PIM	No. Administered PIM	Rationale
Dementia or cognitive impairment (95)	Benzodiazepines	55	32	Avoid due to adverse CNS effects. Avoid antipsychotics for behavioural problems of dementia or delirium unless nonpharmacological options have failed or are not possible and older adult is threatening substantial harm to self or others. Antipsychotics are associated with greater risk of cerebrovascular accident and mortality in persons with dementia.
	H2-receptor antagonists	1	1	
	Antipsychotics, chronic and as-needed use	44	32	
	Other anticholinergics listed in Table 7 (Beers Criteria)	6	4	
	**Total PIMs**	**106**	**69**	
	**Total no. patients (%)**	**56 (58.9)**	**44 (46.3)**	
Falls and Fractures (61)	Anticonvulsants	8	8	Ability to produce ataxia, impaired psychomotor function, syncope, and additional falls; shorter-acting benzodiazepines are not safer than long-acting ones. If one of the drugs must be used, consider reducing use of other CNS-active medications that increase risk of falls and fractures and implement other strategies to reduce fall risk.
	Antipsychotics	12	6	
	Benzodiazepines	30	18	
	TCAs or SSRIs	16	16	
	Opioids	50	31	
	**Total PIMs**	**116**	**79**	
	**Total no. patients (%)**	**51(83.61)**	**38 (62.30)**	
Parkinson’s disease (10)	Metoclopramide or prochlorperazine	2	0	Dopamine receptor antagonists with potential to worsen parkinsonian symptoms. Quetiapine and clozapine appear to be less likely to precipitate worsening of Parkinson disease.
	**Total no. patients (%)**	**2 (100)**	**0**	

Notes: Some patients were prescribed more than one PIM in each category; each PIM was only counted once per patient if it was prescribed both as a regular and PRN medication.

## Discussion

The prevalence of PIMs was quantified in this study which consisted of two groups of older Australians, with the RACF group more likely to be female and older. Women have a longer life expectancy than men^29^ and the difference in age profile between the groups was likely related to admission to an RACF being associated with the need for assistance with activities of daily living.

Although a number of previous studies have examined the prevalence of PIMs in the RACF population *or* in community-dwelling cohorts^30,31^, this study combines data from both settings and compares those data. These findings and their analysis confirm that the use of PIMs is prevalent for older Australians in both settings, placing them at risk of PIM related adverse outcomes.

An earlier Australian study reported that 81.4% of their cohort across 17 RACFs were prescribed a PIM^32^ which is comparable to the present study (83.3%). Of the 4,136 different medications prescribed in this study, 600 were PIMs (14.5%) which is also comparable to the previous study (15.9%)^32^.

Compared to a published systematic review^18^, the prevalence of PIMs in our RACF group (43.2 vs 83.3%) is significantly higher. Morin *et al*. (2016)^18^ also confirmed a trend of increasing PIM usage over time and the results of our study may reflect this trend as our data more closely reflects data published in 2018. Furthermore, Morin *et al*. (2016) also concluded that one of the major contributing factors for increased PIM prescribing was the increasing number of total medications prescribed^18^.

The rate of PIM prescribing in the community setting has been previously described as 20.5% in a 2012 review^10^, 39.8% were prescribed more than one PIM in an Australian study^30^, and a European review of studies that had been published between 2015 and 2018 showed that 22.6% of community dwelling older people had been prescribed a PIM^31^. These findings are all significantly lower than the present study, which indicates that 77.3% of CDOA had been prescribed a PIM, suggestive of increased polypharmacy and inherent PIM prescribing.

Despite no significant difference in mean number of regular medications between the two groups, RACF residents were significantly more likely to be taking three to five PIMs compared with CDOA who were more likely to be only taking one PIM (Table 1). Therefore the risk of being prescribed a greater number of PIMs in this study may be attributable to either a higher absolute number of medications (for example the high number of PRN medications) which was demonstrated in the RACF cohort (31% of total medications were PRN vs. 12% in the community), or to the actual place of residence. However, as noted, many of the PRN PIMs were not actually administered to the RACF residents; hence this is likely an overestimate of the actual risk.

Community dwellers in our study had a higher rate of PPIs prescribed (68.2% vs 58.9% in the RACF cohort, P = <0.01) which is well documented, both in Australia and globally^33–35^. In the Australian setting, the prescribing of PPIs rose by 1318% over 10 years (1996–2006) and in 2006 was 659.8 defined daily doses per 1000 population per day^33^. In that study, the majority of PPIs were prescribed to patients over 80 years of age with 26% of females and 23% of males aged 90–94 years taking a standard dose of PPI daily^33^.

An American study in 2008 identified that the highest distribution of therapeutic category was estrogens, compared with proton pump inhibitors for our study^36^. This difference could be attributed to the changes in prescribing patterns over the years (2000 vs 2017) as prescribing of PPIs has risen significantly during this time period^36^. This is also reflected by other recent international studies^37,38^. The lower prescribing of PPIs in the RACF cohort could be due to increasing awareness of deprescribing leading to a review of PPIs by practitioners working in this setting. However, this may also be due to a difference in the emphasis of therapeutic management in the two cohorts. Due to the unavailability of comorbidity data for the CDOA group, this could not be confirmed.

A significantly greater number of patients were prescribed antipsychotics in the RACF vs CDOA group (36.0% vs 11.8% respectively, P = <0.001). Although this could suggest an over-use of antipsychotics in the RACF setting, it could be indicative of appropriate prescribing for a specific cohort of RACF residents who, due to psychological and behavioural disturbances cannot be appropriately supported in the community. As data were not available for comorbidities for the community dwellers, this could not be confirmed. The high rate of antipsychotic prescribing was largely attributable to risperidone (45 RACF residents vs one community dweller; 14.5% vs 0.45% respectively). The frequency of risperidone used regularly was 14.7% vs 9.7% in residents with and without dementia respectively (data not shown). Risperidone is indicated as first line for behavioural and psychological symptoms of dementia (BPSD) at a dose of 0.25 micrograms twice daily for up to 12 weeks^39^ in Australia. As data were only collected for a 4 week period, it cannot be determined if residents were prescribed this medication within the recommended duration of use. Hence, the medication usage may well have been appropriate.

The overall antipsychotic prescribing rate was also greater in residents with dementia compared with those without, 33.7% and 17.5% respectively (data not shown). This disparity may reflect divergent management of residents with BPSD but it may also indicate a lack of non-pharmacological management, which may be the most appropriate for BPSD including agitation in a non-dementia cohort^40^. This could, however, be related many other issues, such as staffing levels and the severity of dementia. However, these variables were not collected in the RACF sample data.

The overall rate of regular antipsychotic prescription in our study was 25.9% in the RACF residents. This is comparable to another large Australian study which quantified the amount of antipsychotics prescribed in 150 RACFs (amongst 12, 157 residents) at baseline to be 21.6%^41^. Rates of regular benzodiazepine administration in our study for the RACF residents was 29.7%, again similar to the previous study (22.2%)^41^.

Although the prevalence of PRN prescribing of antipsychotics and benzodiazepines was high in the RACF cohort, only 0.6% (2 out of 26 residents prescribed PRN antipsychotics) and 9.3% (24 out of 96 residents prescribed PRN benzodiazepines) were administered these PRN medications. Future studies should investigate this large discrepancy between prescribing and administration rates of these medications.

Although the incidence of PIMs prescribed in the RACF Parkinson’s disease cohort was low, the co-prescription of drugs such as metoclopramide or prochlorperazine could worsen motor symptoms and potentially lead to neuroleptic malignant syndrome^42,43^. Although not administered regularly to any residents during the study period, their prescription does need review for these clients. Pharmacists embedded in RACFs as an integral part of the healthcare team could enable the rationalisation of PRN medications to achieve quality use of medicines^44,45^.

This study also highlights that prescribers will have divergent goals for managing clients in different settings. Overall, the balance of benefit versus harm is likely to be impacted by a person’s place of residence (RACF versus community), as well as current prognosis or morbidity. For example, PPIs were more likely to be prescribed for community dwellers than for those in RACFs, indicative of PPIs being constantly reviewed and de-prescribed in RACFs. In addition, analgesics and laxatives were more commonly prescribed in RACFs, suggesting that symptom management is a high priority in this cohort. In contrast, a greater number of CDOA were prescribed medications for chronic disease management, suggesting a more active treatment paradigm.

Although Table 3 of the Beers Criteria^13^ was used as a more specific tool to identify drug-disease/syndrome interactions in the RACF cohort, not all RACF participants had documented diagnoses in their notes or the diagnoses listed may have not been all inclusive. With no data on diagnoses in the community group, there was no capacity to compare differences in PIM prescribing due to drug-disease/syndrome interactions between the two groups.

As prescribing patterns were only collected over a one month period for the RACF participants, it was not possible to determine if some medications were used for an appropriate time frame. For example, PPIs for <8 weeks and risperidone for BPSD for <12 weeks. Hence, further interpretation of medication appropriateness could not be made.

## Conclusion

One of the barriers identified for hindering deprescribing of PIMs by clinicians is the lack of time and degree of effort to conduct a full medication review^46^. This highlights that regular medication review for all older Australians regardless of their place of residence to minimise PIMs, may be best instigated by pharmacists within RACFs and general practices^44,45^ in collaboration with prescribers. Pharmacist-driven medication reviews have been shown to decrease the number of PIMs prescribed in geriatric primary care and residential aged care settings^47,48^. Other Australian studies have also demonstrated that once drug related problems are identified by pharmacists, there is a high uptake of medication change by prescribers across aged care and general practice settings^49,50^.
